# Supplementary material for: Complex‐centric proteome profiling by SEC‐SWATH‐MS
Source: Mol Syst Biol. 2019 Jan 14;15(1):e8438. doi: 10.15252/msb.20188438 (PMC6346213; doi:10.15252/msb.20188438)
Supplement: Supplementary file 8 — Dataset EV7 [file MSB-15-e8438-s008.zip › feature_plots_string/Q10713.pdf]

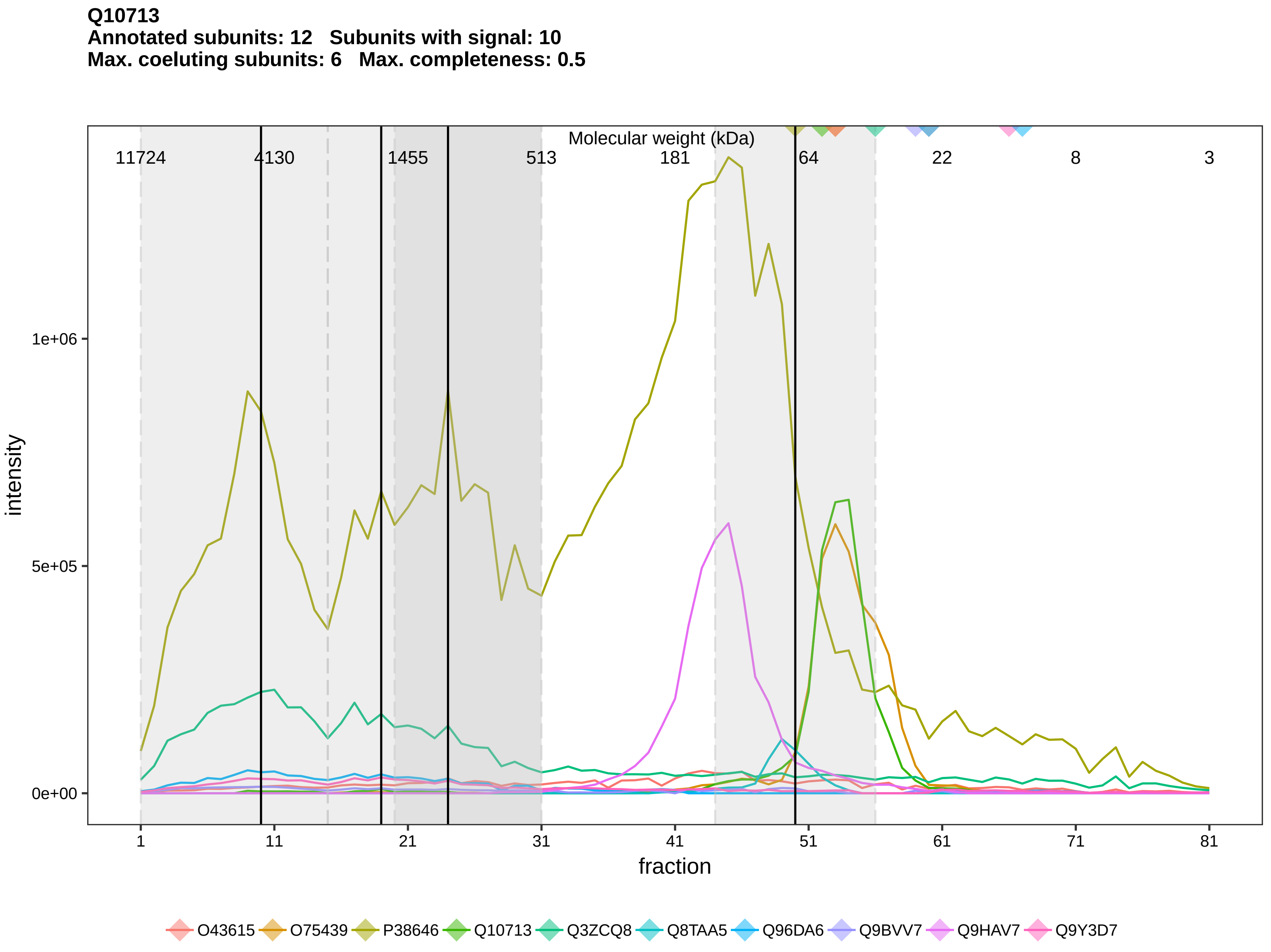
Q10713
Annotated subunits: 12 Subunits with signal: 10
Max. coeluting subunits: 6 Max. completeness: 0.5
Molecular weight (kDa)
11724
4130
1455
513
181
64
22
8
3
intensity
1e+06
5e+05
0e+00
1
11
21
31
41
51
61
71
81
fraction
O43615
O75439
P38646
Q10713
Q3ZCQ8
Q8TAA5
Q96DA6
Q9BVV7
Q9HAV7
Q9Y3D7
